# Roles of the MYST Family in the Pathogenesis of Alzheimer’s Disease via Histone or Non-histone Acetylation

**DOI:** 10.14336/AD.2020.0329

**Published:** 2021-02-01

**Authors:** Yuhong Li, Hui Huang, Man Zhu, Hua Bai, Xiaowei Huang

**Affiliations:** ^1^State Key Lab for Conservation and Utilization of Bio-Resources, Yunnan University, Kunming, Yunnan, China.; ^2^Yunnan Institute of Tropical Crops, Jinghong, China; ^3^College of Public Health, Kunming Medical University, Kunming, China

**Keywords:** Alzheimer’s disease, MYST, Epigenetic, MOZ, MORF, HBO1, hMOF

## Abstract

Alzheimer's disease (AD) is one of the most common neurodegenerative diseases and a major cause of death among elderly individuals. The etiology of AD involves a combination of genetic, environmental, and lifestyle factors. A number of epigenetic alterations in AD have recently been reported; for example, studies have found an increase in histone acetylation in patients with AD and the protective function of histone deacetylase inhibitors. The histone acetylases in the MYST family are involved in a number of key nuclear processes, such as gene-specific transcriptional regulation, DNA replication, and DNA damage response. Therefore, it is not surprising that they contribute to epigenetic regulation as an intermediary between genetic and environmental factors. MYST proteins also exert acetylation activity on non-histone proteins that are closely associated with the pathogenesis of AD. In this review, we summarized the current understanding of the roles of MYST acetyltransferases in physiological functions and pathological processes related to AD. Additionally, using published RNA-seq, ChIP-seq, and ChIP-chip data, we identified enriched pathways to further evaluate the correlation between MYST and AD. The recent research described in this review supports the importance of epigenetic modifications and the MYST family in AD, providing a basis for future functional studies.

## Alzheimer's disease

Alzheimer's disease (AD) was first discovered in 1906 and is now considered the main cause of dementia, making it a major issue in the context of the acceleration of aging in the population. As a primary degenerative encephalopathy, its clinical manifestations include a decline in the memory functions in the early stage and a gradual, complete loss of cognitive ability, speech, and even a normal behavioral function. Since the disease course has been thought to be irreversible, AD is an intractable medical problem and a major social burden. Since the year 2000, the mortality rate for AD has increased by 89% while that for heart diseases has decreased by 14% during the same period. As this trend continues, it is estimated that the total medical investment in AD may reach 1.1 trillion dollars by the year 2050 in the United States alone [1].

The characteristic pathological changes in most patients with AD are consistent and include β-amyloid deposition, entanglement of the neurogenic fibers, senile plaque formation, reduction in the number of memory neurons, and atrophy of the cerebral cortex. However, the etiology involves a combination of genetic, environmental, and lifestyle factors. At the genetic level, mutations in genes encoding the amyloid precursor protein (APP) and presenilins 1 and 2 have been detected in the autosomal dominant form of AD. However, autosomal dominance accounts for only a small proportion (about 5%) of cases. Among sporadic AD, the ε4 allele of *APOE* is the greatest risk factor, responsible for about 30%-35% of cases [2]. Various environmental inputs, such as pesticide exposure, a high-fat diet, and a sedentary lifestyle, have been hypothesized to remodel the epigenome and thus are closely related to the occurrence of AD. For example, the incidence of degeneration of the frontotemporal lobe increases with age along with an excessive production of ROS and decreased cellular antioxidant defenses [2]. ROS accumulation can alter DNA methylation and histone acetylation, thereby influencing the amyloid-β production [3]. Accordingly, epigenetic changes in the expression of pathogenic genes have been suggested to be another major determinant of AD development [4].

## Histone acetylases

Histone acetylation, one of the main types of histone modification, involves the addition of an acetyl group to lysine residues in the N-terminal tail or on the surface of the nucleosome core of histones. Acetylated histones usually relax the chromatin structure, thereby increasing transcription. Acetylation also affects the replication and repair of DNA [5]. This modification is commonly performed by histone acetyltransferases (HATs) at specific lysine sites of histones H2A, H2B, H3, and H4. HATs have been identified in a variety of eukaryotic organisms from yeast to humans and are classified into three primary subclasses according to a structural homology: (1) the GNAT (Gcn5-related *N*-acetylase) family, containing Gnc5, PCAF, and the elongator complex protein 3 (ELP3); (2) the MYST family, consisting of Tip60, MOZ/MYST3, MORF/MYST4, HBO1/MYST2, and hMOF/MYST1; and (3) the p300/CBP (CREB-binding protein) family, including p300 and CBP [6].

Direct links between HATs and AD have been established; there is an evidence of the increased histone acetylation in AD and protective functions of the histone deacetylase inhibitors [7]. In particular, the roles of the GNAT and p300/CBP families in AD have been demonstrated. H2B and H4 acetylation in the rat dorsal hippocampus produced by PCAF, CBP, and p300 may enhance the expression of memory/plasticity-related genes and therefore be helpful in consolidating a spatial memory. However, PCAF selectively acetylates NF-κB at Lys-122, which triggers the inflammatory response induced by Aβ in mammalian cells [8, 9]. The p300/CBP family exhibits a neuroprotective activity in the context of AD development, and therefore, loss or over-expression of CBP/p300 contributes to the neuronal death [10, 11]. In addition to a p300-mediated histone acetylation, the acetyltransferase activity of p300 influences the hyperacetylation of the tau proteins in AD brains [12].

In addition to the direct roles of HATs, there is an evidence of additional indirect effects in AD. For example, GCN5 acetylates CDK5 at Lys33, which affects the kinase activity [13]. Cdk5 activation is responsible for abnormalities of the amyloid, tau, and neurofilament proteins in AD, implying that GCN5 may be critically involved in the pathogenesis of AD, but additional experimental evidence is needed [14, 15, 16].

Here, we reviewed recent evidence of the key roles of epigenetic mechanisms in AD with a focus on the MYST family. Furthermore, we utilized publicly available data from high-throughput genomic assays to clarify the mechanisms by which the MYST proteins contribute to the pathogenesis of the disease.

## The MYST family and AD

The MYST family currently includes five human HATs, Tip60, MOZ, MORF, HBO1, and MOF, and orthologs have been detected in other eukaryotes. They function in multi-subunit protein complexes with evolutionarily conserved compositions. With respect to the structure, MYST acetyltransferases have a distinct histone acetyltransferase domain, which contains a C2HC zinc finger and an acetyl-CoA-binding site [17]. Through a MYST-dependent chromatin acetylation, the MYST family regulates a variety of cellular processes, ranging from gene regulation to DNA repair, cell cycle, stem cell homeostasis, and development. Non-chromatin substrates of MYST enzymes and a few cellular pathways independent of a MYST-mediated histone acetylation have recently been reported, emphasizing the multifunctional nature of this highly conserved acetyltransferase family. Different types of MYST proteins have distinct physiological functions and roles in AD as summarized below.

## Tip60 (KAT5)

**Figure 1.**
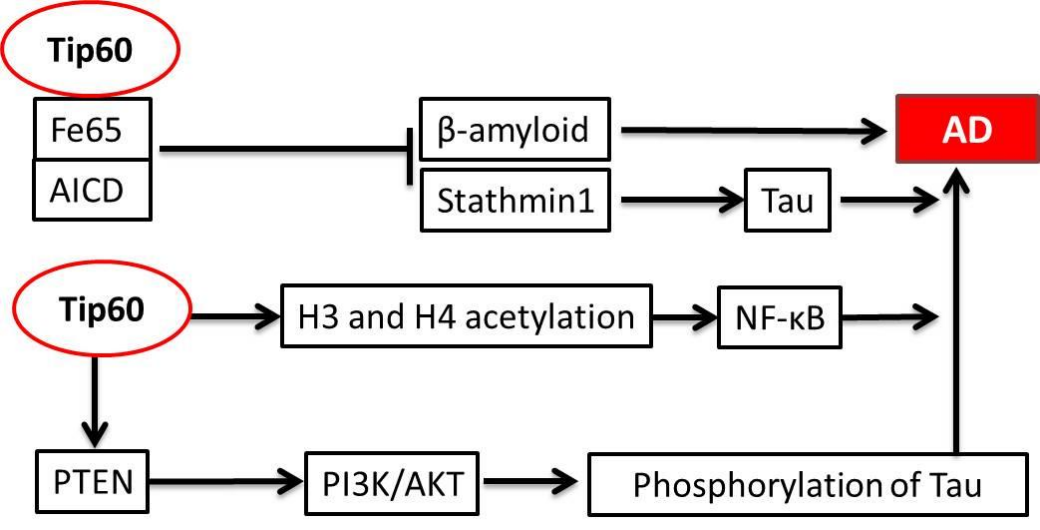
Summarized diagram of Tip60 in AD. First, the complex composed of Fe65, AICD, and Tip60 can inhibit β-amyloid and Stathmin1 while Stathmin1 can promote tau phosphorylation. At the same time, Tip60 directly acetylates the H3/H4 histones and activates PTEN, thereby affecting the inflammatory pathogenesis of AD and phosphorylation of tau, respectively.

TIP60 (Tat-interactive protein 60) functions as a key regulator of homeostasis via the regulation of gene transcription, DNA repair, and apoptosis with target genes such as the ATM protein kinase and p53 tumor suppressor [18]. Its roles in AD have been investigated most intensively among members of the MYST family and have been summarized in Figure 1 [19]. Tip60 directly affects the amyloid-β precursor protein (APP) and the tau protein. In the pathological changes of AD, Fe65 (a ligand of APP) binds to the intracellular subdomain of APP (AICD), and their interaction in the nervous system significantly increases the transfer of APP to the cell surface as well as the accumulation of β-amyloid peptides in vitro [20]. However, a recent study has shown that Tip60 forms a multimeric complex with Fe65 and AICD in the cytoplasm, thereby stimulating gene transcription via recruitment of a Tip60-dependent histone acetylation [21, 22]. Studies of cells co-expressing AICD/FE65/TIP60 have also shown that the expression of AICD as well as the complex downregulates Stathmin1, a cytoskeleton-associated protein, and therefore disrupts microtubule dynamics and influences the neurofibrillary tangles in AD [23, 24]. In addition, the co-localization and binding of Fe65 and the tau protein in neurofibrillary tangles of the hippocampus suggest that Fe65 plays an additional role in the crosslinking between the β-amyloid peptides and neurofibrillary tangles in AD [25, 26]. However, the involvement of Tip60 in the above-mentioned pathological process requires further elucidation.

Studies of Tip60 have provided substantial insights into cognitive disorders and epigenetics. Experimental data generated by Xu and colleagues have shown that a steady cognitive deterioration and the corresponding accumulation of β-amyloid peptides are effectively blocked or even rescued by increasing the Tip60 levels in the mushroom body of *Drosophila* [27]. To determine the molecular mechanisms underlying the neuroprotective function of Tip60, ChIP-seq analysis has identified a variety of cognitive genes that are dysregulated in Tip60-mutant flies [28]. Additionally, environmental enrichment has been hypothesized to improve the cognitive ability in neuropathic diseases, and Tip60 may mediate these beneficial effects [29].

In addition to its direct interactions with APP, thereby improving the cognitive ability and conferring neuroprotection, Tip60 may be involved in the regulation of other AD-related biological processes. For example, Tip60 positively modulates the PTEN levels and subsequently activates PI3K/AKT in cholangiocarcinoma tissues [30, 31]. However, an earlier report showed that AKT contributed to the phosphorylation of tau at Thr212 and Ser214 and was a risk factor for AD [30]. Additionally, Tip60 directly catalyzes H3 and H4 acetylation in the promoter regions of the NF-κB target genes (e.g., *IL-6, IL-8, XIAP*, and *cIAP*) and maintains levels of acetylated Lys-310 of RelA/p65 [32]. NF-κB is also widely appreciated as a key gene in the inflammatory pathogenesis of AD (Fig. 1).

**Figure 2.**
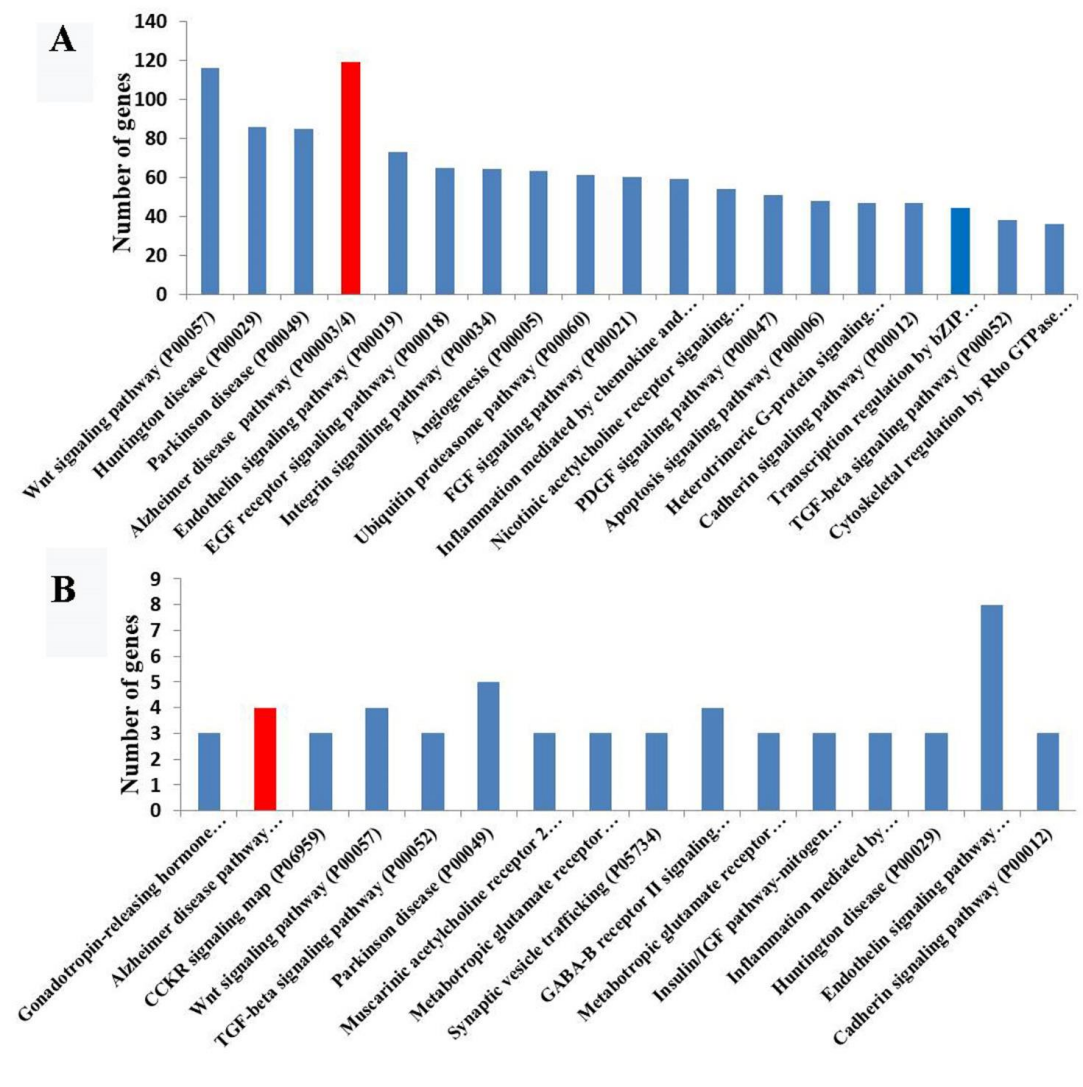
Tip60-mediated target genes involved in AD. PANTHER pathway analyses of the differentially expressed genes determined by ChIP-seq using a Tip60 antibody in *Drosophila melanogaster* (A) and determined by RNA-seq in Tip60-mutant *Drosophila melanogaster* (B). The 20 most highly enriched pathway entries have been displayed.

Using ChIP-seq data for Tip60 (GSE76612) and RNA-seq data for the Tip60-related genes generated by Xu and colleagues, we performed a PANTHER pathway analysis (http://pantherdb.org/; http://kobas.cbi.pku.edu.cn/anno_iden.php) and identified enrichment for the AD-presenilin pathway (P00004), AD-amyloid secretase pathway (P00003), and a series of AD-related pathways in these two datasets (Fig. 2A and 2B) that were consistent with previous findings.

## MOZ/MYST-3 (KAT6A) AND MORF/MYST4 (KAT6B)

**Figure 3.**
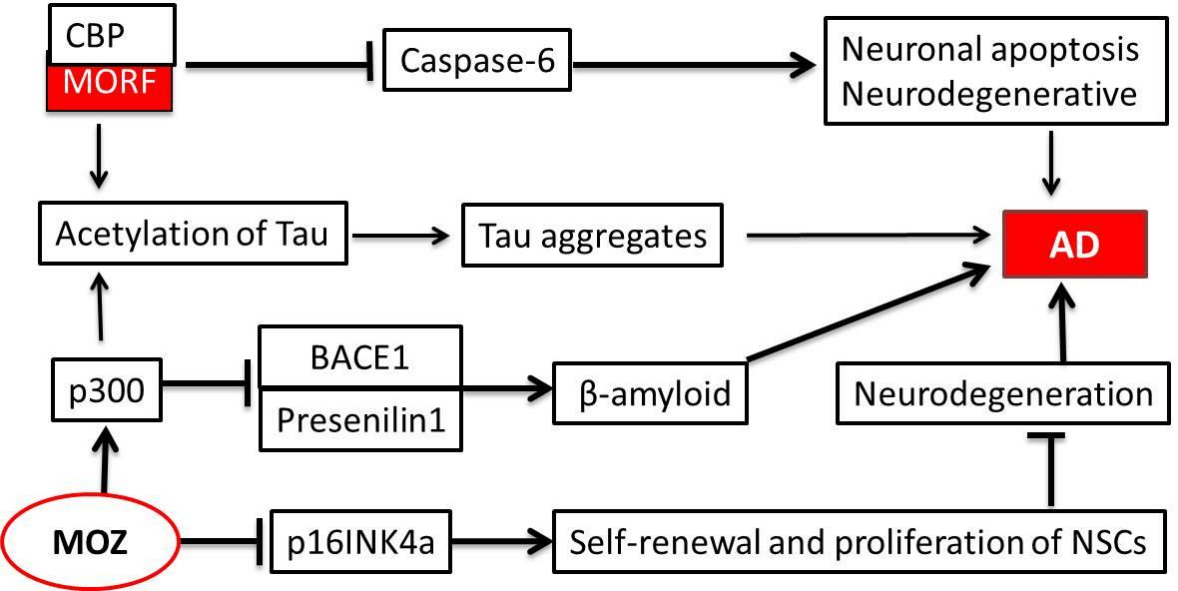
Involvement of MOZ and MORF in AD. MOZ binds to the promoter of p16INK4a and silences the p16INK4a expression, which in turn inhibits the self-renewal and proliferation of NSCs. The interaction of MOZ/p300 can acetylate tau or suppress the expression of Presenilin1 and BACE1. Additionally, the fusion genes of MORF and CBP can aggravate the pathogenesis of AD by either inhibiting the expression of the neuronal apoptosis factor (caspase-6) or promoting the abnormal accumulation of acetylated tau.

KAT6A, also known as MOZ (monocytic leukemia zinc finger protein) or MYST3, has crucial functions in the long-term reproduction of hematopoietic stem cells and the formation of memory B cells [33]. Although MOZ seems to indirectly affect AD, a few studies have shown that it is involved in the self-renewal of neural stem cells (NSCs) and is a potential therapeutic target for AD [34, 35, 36]. MOZ can silence the p16^INK4a^ expression by binding to the promoter of p16^INK4a^, which is critical for the self-renewal and proliferation of NSCs [37]. Increased neuronal proliferation may lead to the attenuation of an Aβ-induced neurodegeneration in an AD mouse model [38]. Furthermore, the zinc finger and acetyltransferase domains of MOZ fuse to the gene encoding the E1A-associated protein p300 (p300) at t (8; 22) (p11; q13) in acute monocytic leukemia based on FISH experiments [39, 40]. The role of p300 in AD is supported by an experimental evidence that the addition of a p300 inhibitor significantly suppresses the expression of presenilin 1 (PS1) and the beta-site amyloid precursor protein cleaving enzyme 1 (BACE1) via a reduced H3 acetylation within their promoter regions [41, 42]. In addition, p300 has an effect on the p300-mediated acetylation of tau in a concentration-dependent manner and in turn represses the tau assembly [43, 44], suggesting that MOZ/p300 influences the aggregation of tau (Fig. 3).

MORF/MYST4 (monocytic leukemia zinc finger protein-related factor), also known as KAT6B, is highly homologous to MOZ/MYST3 (KAT6A) and preferentially acetylates H2A, H3, and H4 in vitro [45]. Interestingly, MORF/MYST4 (KAT6B) is also involved in acute myeloid leukemia and the myelodysplastic syndrome with fusion to the CREB-binding protein (CBP) instead of p300, similar to that exhibited by MYST3/KAT6A [46]. In fact, CBP and p300 are highly homologous (with an overall identity of > 70%) and have highly overlapping functions via their interactions with similar partners and substrates [47]. Similar to the HATs described above, CBP/p300 in the neural system can confer neuroprotective functions and their aberrant expression leads to neuronal apoptosis and neurodegenerative diseases via a caspase-6 activation [10, 11, 47]. The acetylation of tau at lys28 via CBP/p300 impairs the interaction between tau and microtubules, contributing to the formation of insoluble tau aggregates in AD [48] (Figure 3). However, the involvement of MOZ/MYST3 (KAT6A) or MORF/MYST4 (KAT6B) in the effects of CBP/p300 remains unclear.

**Figure 4.**
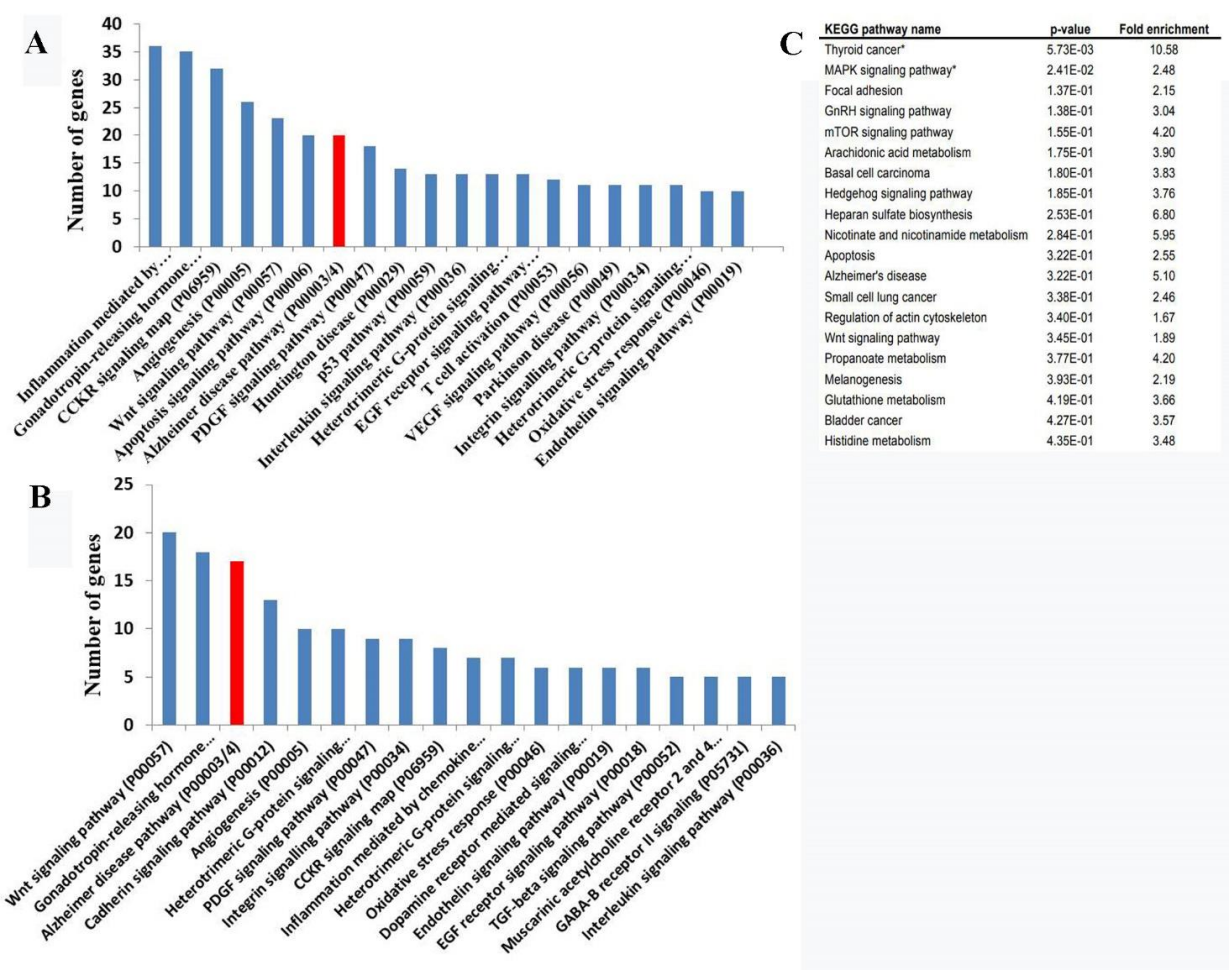
MOZ-mediated target genes involved in AD. PANTHER pathway analyses of the differentially expressed genes in leukemic stem cells with the MOZ-TIF2 fusion gene (A), MOZ-knockdown in SUM-52 cells (B), and siRNA knockdown of MORF in the HEK 293 and HeLa cells (C). The 20 most highly enriched pathway entries have been displayed.

We also performed a PANTHER pathway analysis using RNA-seq data of the *MOZ-TIF2* fusion gene in leukemic stem cells (Figure 4A), MOZ knockdown in SUM-52 cells (Figure 4B), and MORF knockdown in HEK293 and HeLa cells (Figure 4C). Our results illustrated enrichment for the AD-presenilin pathway (P00004) and the AD-amyloid secretase pathway (P00003) in all datasets.

## HBO1/MYST-2 (KAT7)

HBO1 (histone acetyltransferase binding to ORC1) interacts with ING4 and BRPF3 to acetylate H4 at lysines 5, 8, 12, and 14, thereby activating gene transcription. HBO1 also functions as a co-activator of the DNA pre-replication complex formation based on a genome-wide analysis showing high enrichment for HBO1, BRPF3, and H3K14ac at ORC1-binding sites and replication origins [49]. A few indirect correlations between HBO1 and AD have been inferred. For example, HBO1 increases the activity of β-catenin in the Wnt signaling pathway in both the J82 and T24 cells [50], and either a reduced β-catenin in AD or the Wnt inhibitor Dkk1 (Dickkopf-1) positively regulates tau phosphorylation and exacerbates the cognitive deficits, establishing a link between HBO1 and AD [51, 52].

**Figure 5.**
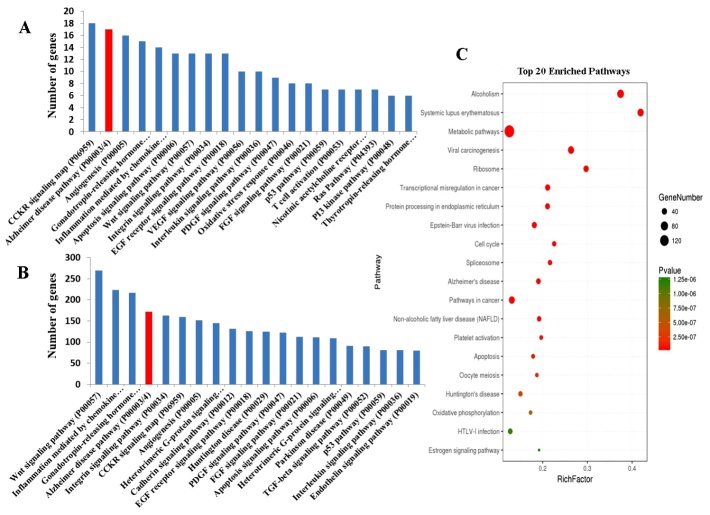
HBO1-mediated target genes involved in AD. PANTHER pathway analyses based on ChIP-seq data obtained using an HBO1 antibody (A), ChIP-chip data using HBO1 antibodies in K562 cells (B), and a pathway enrichment analysis of ChIP-chip data for K562 cells (C). The 20 most highly enriched pathway entries have been displayed.

To determine the roles of HBO1 in AD, ChIP-seq and ChIP-chip data for human cell lines were analyzed to identify target genes that bind to HBO1 [49, 53]. The PANTHER pathway (Figures 5A and 5B) and pathway enrichment analyses (Figure 5C) showed that targets of HBO1 included genes associated with neurodegenerative diseases, such as AD, HD (Huntington’s disease), and PD (Parkinson’s disease).

## MOF/MYST1 (KAT8)

**Figure 6.**
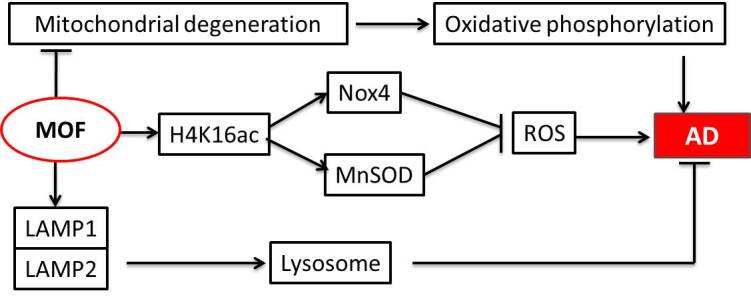
The roles of MOF in AD. MOF can inhibit the degeneration of mitochondria and decrease AD induced by an oxidative phosphorylation. Acetylating H4K16 and MOF also activates Nox4 and MnSOD as well as decreases ROS. Finally, MOF activates lysosomes via LAMP1 and LAMP2.

MOF (males absent on the first) usually functions as a catalytic subunit in acetyltransferase complexes. In human cells, it can form two different protein complexes (MSL and NSL), both of which acetylate histone H4 at lysine 16 (H4K16). However, the NSL complex has wider substrate specificities with histone H4 acetylated at lysine 5 and 8 (H4K5 and H4K8) [54, 55]. Functional studies have evaluated the direct correlation between the MOF complex and the pathogenesis of AD, revealing that the complex regulates a series of cellular processes or pathways that participate in the development of AD. There is substantial evidence that the accumulation of reactive oxygen species (ROS), deleterious effects on biomolecules (e.g., proteins, DNA/RNA, and lipids) from an oxidative stress, mitochondrial dysfunction directly or indirectly caused by ROS, and an eventual apoptosis or necrosis of neurons contribute to the etiology of AD [56, 57]. Silencing or overexpression of MOF may downregulate the ROS-generating gene *Nox4* or enhance catalase and MnSOD dependent on the activity of MOF histone acetyltransferase and the level of H4K16, respectively [58]. Consistent with these findings, RNA-seq data for the MOF deficiency mutant showed a significant downregulation of antioxidant genes, leading to an increased production of ROS [59]. As a dual-transcriptional regulator of the nuclear and the mitochondrial genomes connecting epigenetics and metabolism, MOF knockout leads to a mitochondrial degeneration, disturbance of the mitochondrial nutrient metabolism, and deregulation of the oxidative phosphorylation pathways [60]. Lysosomes also play an important role in AD. On one hand, Aβ impairs the lysosomal function via lysosomal de-acidification and membrane permeabilization. On the other hand, the levels of the lysosomal proteins, such as LAMP1 (lysosome-associated membrane protein 1) and LAMP2, the important two markers of lysosomal function, change with the occurrence of Aβ deposition or AD [61, 62]. A genome-wide expression analysis further showed that the histone acetyltransferase MOF was able to maintain the transcription of the lysosome-related genes [63] (Figure 6).

**Figure 7.**
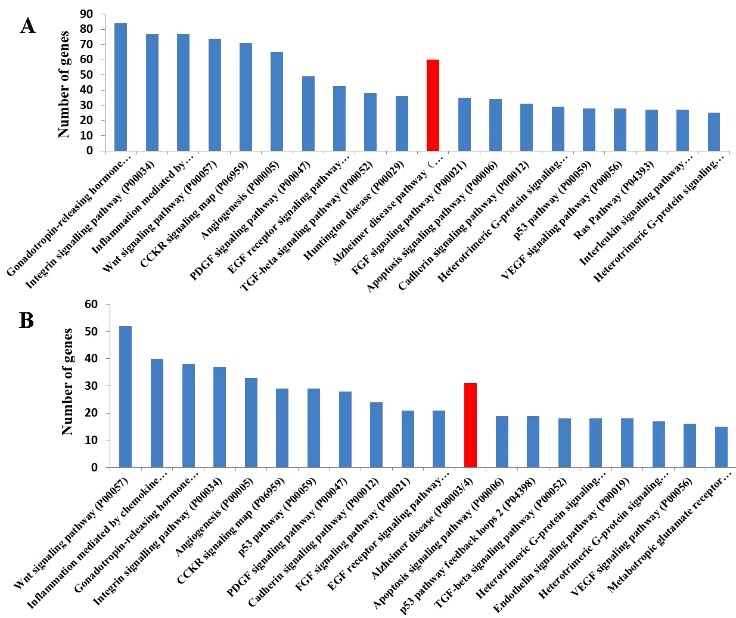
MOF-mediated target genes involved in AD. PANTHER pathway analysis of ChIP-seq data using an MOF antibody in the embryonic stem cells (A), and genes that were significantly altered in the MOF-deleted mouse embryonic fibroblasts (B). The 20 most highly enriched pathway entries have been displayed.

To establish the direct link between MOF and AD, we performed an additional PANTHER pathway analysis based on ChIP-seq data for MOF-binding motifs in the embryonic stem cells [64] (Figure 7A) and PANTHER pathway analyses of the genes that were significantly altered in the MOF-deleted mouse embryonic fibroblasts [63] (Figure 7B). Various genes associated with the pathogenesis of AD as well as the AD-related cellular processes were identified.

## Conclusions

Studies of the relationships between HATs and neurodegenerative diseases have revealed that the MYST family has direct effects on AD, PD, and HD. However, partial relationships among them are mediated by intermediate cellular processes or genes. For example, the Wnt signaling plays critical roles in several cellular processes, including synaptic activity, pre- and postsynaptic assembly, function at the glutamatergic synapses, neurogenesis, and neuroprotective memory consolidation. Furthermore, TGF-β, apoptosis, and/or oxidative stress response pathways with well-established roles in AD are targets of MYST. Our analysis of publicly available high-throughput data supports these previous findings and provides additional insight into the mechanisms by which this protein family contributes to AD. Collectively, additional experimental studies of targets of TIP60, MOZ, HBO1, and MOF as well as direct and indirect contributors to AD will be needed to validate their roles.
